# Respiratory Monitoring by a Field Ionization Sensor Based on Trichel Pulses

**DOI:** 10.3390/s140610381

**Published:** 2014-06-12

**Authors:** Fucheng Deng, Lingyun Ye, Kaichen Song

**Affiliations:** College of Biomedical Engineering and Instrument Science, Zhejiang University, Hangzhou 310027, China; E-Mails: dunfen@zju.edu.cn (F.D.); kcsong@zju.edu.cn (K.S.)

**Keywords:** respiratory monitoring, Trichel pulses, field ionization sensor

## Abstract

In this paper, a novel method for respiratory monitoring is presented. The method is based on Trichel pulses (TPs) using a simple field ionization sensor which consists of a needle electrode and a plate electrode. Experiments have been conducted to demonstrate that different respiratory patterns, including normal, ultra-fast, deep breaths, and apnea could be easily monitored in real time by detecting the changes in the TP frequency. The vital capacity could also be assessed by calculating the variation of TP frequency. It is found that the operation principle of the proposed sensor is based on the effects of breath airflow and the atomized water in exhaled air on the TP frequency by changing the ionization process and the dynamics of charged particles in the short gap. The influences of applied voltage and ambient parameters have also been investigated.

## 1. Introduction

With the increase in life expectancy and ageing population, as well as the constantly growth of chronic diseases, there has been a great need for in-home health care devices to monitor vital signs such as pulse, blood pressure, respiration and electrocardiography (ECG) patterns [1,2]. Good sensors or monitoring systems with high sensitivity, fast response, low cost, reliability and comfort can be of great help for the popularization of in-home health care devices and the enhancement of life quality for the users.

There are currently many approaches for respiratory measurement. Pressure sensors based on pressure-sensitive elements are widely used in medical field [3]. A microwave sensor for non-contact measurement of respiratory tidal volume has been reported [4]. Similarly, ultra-wideband radar has been used for tracking the chest wall movement of a human subject to detect sleep apnea and estimate breathing rate [5]. Piezoresistive and capacitive-type MEMS flow sensors have been developed based on momentum transfer of the mass flow [6,7]. Humidity-type sensors are typically implemented for breath sensing with a sensitive film or layer such as polyimide, alumina, and porphyrin [8,9]. Carbon nanotubes (CNTs) were also used to develop breath sensors and biosensors [10–12]. A new body sensor for the measurement of respiration rate based on the amplitude changes in the body surface potential differences between two proximal body electrodes has been proposed [13]. Most of these approaches can assess single respiratory parameter, while ideal breath sensors should provide more information about respiration [9,14]. In order to build ideal breath sensors, demands for novel methods still exist.

Since a miniaturized gas ionization sensor using CNTs was reported by Modi *et al.* [15], many studies on field ionization sensors have been performed [16–19]. Gas ionization sensors work by fingerprinting the ionization characteristics of gases [15]. The prebreakdown current or voltage was usually measured as the sensing signal, but the current was very small and on the order of pA, which was not very easy to measure. Much effort has been done to extensively lower the external voltage for the sensor making it suitable for low voltage electronics integration [20–22]. Our goal is to develop a simple low-cost breath sensor with good performance for in-home use. In this paper, we propose a method of respiratory monitoring by field ionization sensor based on Trichel pulses (TPs). TP is an interesting phenomenon of weak partial discharge which is characterized as highly regular current pulses. Respiration can be easily monitored by the measurement of repetition frequency rather than the amplitude of breakdown current. The TPs-based breath sensor is very simple and only requires a sharp electrode and a plate electrode with a constant voltage applied to them. There is no need for particular materials or any complicated manufacturing.

## 2. Mechanism and Experimental Setup

### 2.1. Sensing Mechanism

The phenomenon of TPs was first studied systematically by Trichel [23] and later reviewed by Loeb [24,25]. Many theoretical and numerical studies have been performed to explain the development of TPs in the last three decades [26–30]. It is believed that TPs usually take place in electronegative gases, e.g., air and oxygen. The main ionization process is restricted in a very small area close to the tip where the electric field is extremely intense due to the small radius. In other words, only the cathode with its sharp tip plays a great role while the anode plays a minor role in the discharge. The negative ions are formed by attachment when the electrons move towards the anode. As the velocity of ions is relatively small compared with that of electrons, space charges, especially negative ions, accumulate in the discharge gap and suppress the electric field in the critical ionization area. The ionization process is terminated and cannot recover until the negative ion cloud moves away. It is the periodic termination that leads to the formation of regular TPs [23].

The air flow could have an effect on the movement of negative ion clouds by changing the dynamics of negative ions. When the air flow was perpendicular to the symmetry line between the tip and plate, the TP frequency would decrease with the increasing flow velocity [31]. A numerical model has been proposed to explain the mechanism and more details can be found in our previous work [32]. In short, when the breath air flow acts on the negative ions by imparting momentum in the discharge gap, the movement of the negative ion cloud towards the anode is slowed down, leading to the decrease in TP frequency. If human exhaled breath air acts on the ions periodically, the TP frequency reduces periodically too. That's how the respiration rate can be monitored.

However, the breath air flow does not totally account for the high sensitivity of the sensor for respiration, as will be shown later. The composition of exhaled breath, e.g., water, also plays an important role. It will be demonstrated later that the atomized water in exhaled air reduces the TP frequency substantially. It is supposed that the atomized water tends to attach electrons more efficiently and moves much slower due to its large weight. Thus, the recovery of the electric field is much slower and the TP frequency is significantly reduced.

### 2.2. Experimental Setup

**Figure 1.**
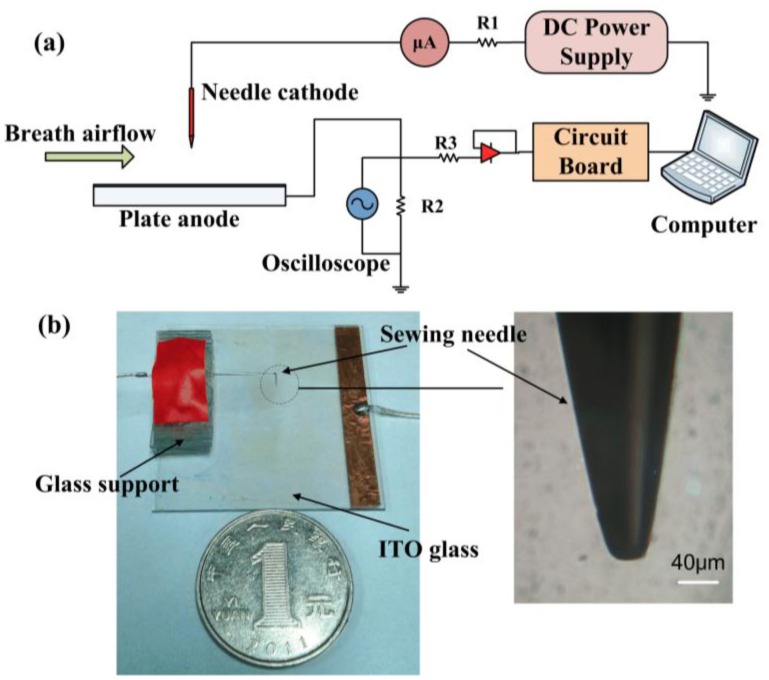
Overview of the experimental setup. (**a**) Schematic diagram for the testing platform; (**b**) The practical needle-to-plate configuration and the morphology of needle tip.

Figure 1 depicts an overview of the experimental scheme. An inexpensive commercial sewing needle made of steel was used as the cathode. The curvature radius of the tip was measured with a microscope and the value was about 30 μm. The anode was a squared plate made of common indium-tin oxide (ITO) glass with a length of 30 mm. As a matter of fact, materials for the electrodes do not matter. It will be fine with a sharp electrode and a plate electrode. A dc power supply (RR10-2R/DDPW, GAMMA, Ormond Beach, FL, USA) was used to apply a constant negative voltage to the cathode. The anode was grounded through a resistor. The average discharge current was measured by an ammeter. The waveform of TPs could be monitored with an oscilloscope (Infiniium 54832D MSO, Agilent, Santa Clara, CA, USA). A circuit based on a microcontroller (TMS320F2810, manufacturer, TI, Dallas, TX, USA) has been designed for the measurement. The discharge current pulse was firstly amplified with commercial amplifiers and then regulated into square wave signal with a Schmitt-trigger inverter. The microcontroller calculated the frequency of the signal and finally uploaded the results to a computer. The circuit was not complex and could be easily implemented with low-cost commercial electronic components. In the experiments, the update rate of measurement was 50 Hz.

**Figure 2.**
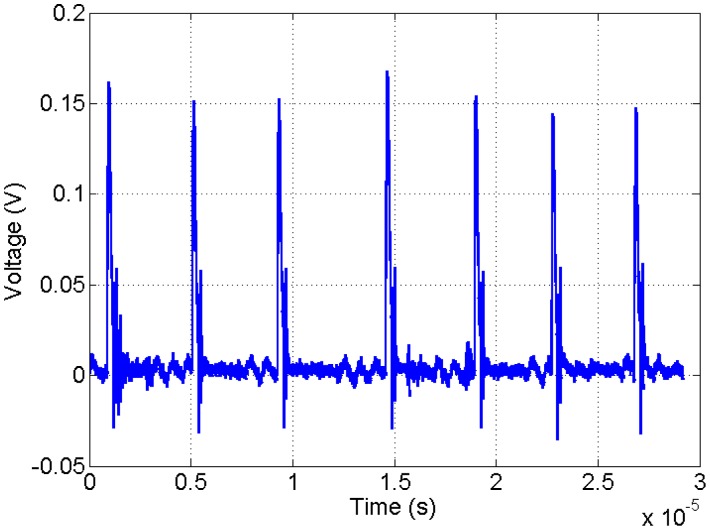
Typical Trichel pulse waveforms.

The experimental environments were set under room temperature, atmosphere pressure, and relative humidity. The needle-to-plate (NTP) structure was placed in open air when the breath experiments were conducted. The air gap was about 4.5 mm and kept unchanged in all the experiments. No current pulses would be detected until the applied voltage grew to the threshold when the ionization process was intense enough to keep the discharge self-sustained. The regular TPs would build up rapidly once the applied voltage was over the threshold. The typical waveform of TPs is shown in Figure 2. A single TP has sharp edges. The pulse width is in the order of 10 ns. The observed duration is limited by the electrical characteristics of the discharge circuit and could be shorter [25]. The average current is small and in the order of 10 μA. The TPs signal was input to the circuit and processed. The repetition frequency was measured in real time. It will be shown later that the baseline frequency can be changed with the applied voltage. However, in the respiration experiments, the applied voltage was kept unchanged and the baseline frequency was about 250 kHz. The variation of TP frequency with the respiration was always the focus.

## 3. Results and Discussion

### 3.1. Respiratory Rate Assessment

**Figure 3.**
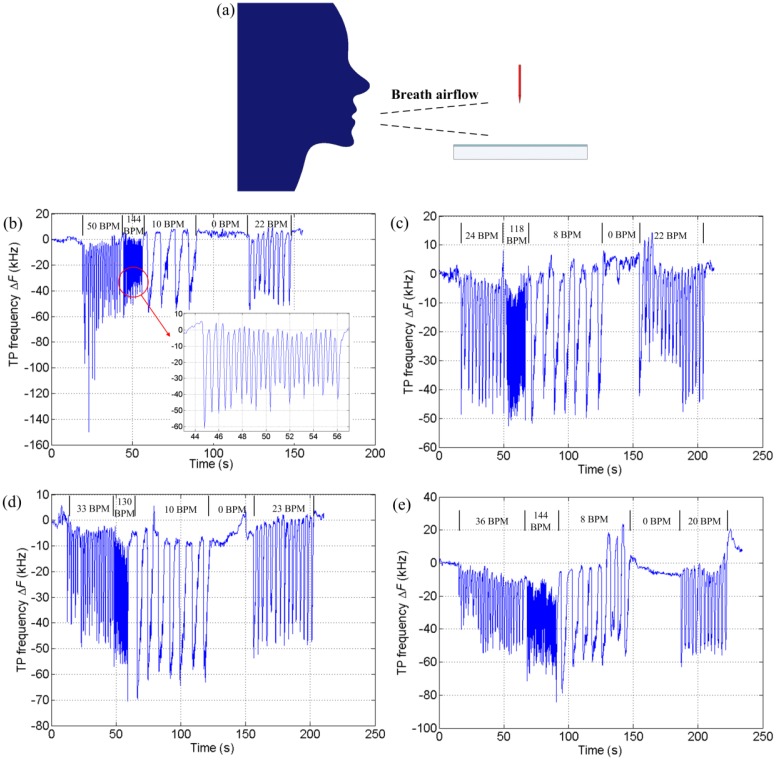
(**a**) Respiratory schematic diagram; (**b**) Variations of TP frequency with different patterns of consecutive breath for subject No. 1; (**c**) For subject No. 2; (**d**) For subject No. 3; (**e**) For subject No. 4.

Healthy volunteers were recruited for the respiratory monitoring experiments. They were required to take breaths consecutively according to the instructions in order to simulate different breath patterns in everyday life such as normal breath, rapid breath, deep breath, and apnea. The subjects took breaths without a mask or mouthpiece in front of the NTP. The distance between the subjects' mouth and the NTP was kept at about 20 cm for consistency, as is shown in Figure 3a. Four subjects' respiratory tests are shown in Figure 3b–e. The respiratory rate, in breaths per minute (BPM), was assessed by calculating the number of pulses per unit time. It can be seen that respiratory rates for different patterns, the normal or abnormal, can be easily detected. The response of the TPs-based sensor is very fast. As fast as 144 BPM of rapid breath can also be readily measured in real time as the inset of Figure 3b shows. The TP frequency rises up to the baseline rapidly once the respiration has stopped. What accounts for the fast response is the avalanche ionization process in the discharge gap which is extraordinarily fast. This is different from conventional method such as absorption/desorption-type, capacitive-type, and movement-based.

### 3.2. Vital Capacity Assessment

**Figure 4.**
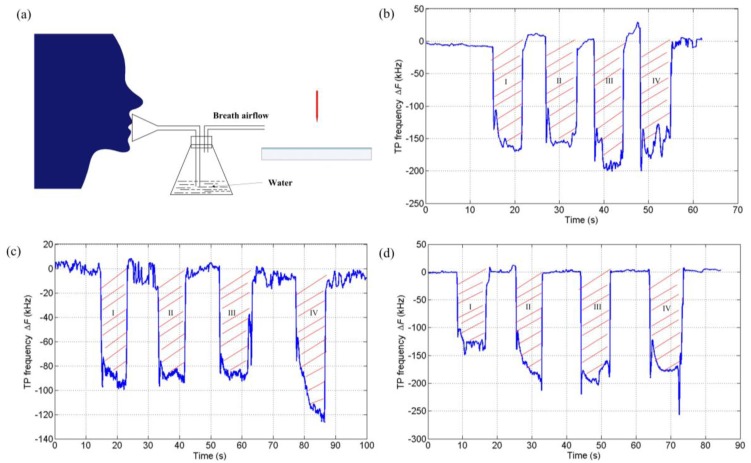
(**a**) Experiment scheme for the vital capacity assessment; (**b**) Variations of TP frequency for VC testing for subject No. 1; (**c**) For subject No. 2; (**d**) For subject No. 3.

It can be noticed from Figure 3 that the detailed responses of the sensor for different subjects are different. More information about the respiration, e.g., the strength, tidal volume, even the breath composition, can be extracted from the variation curve of TP frequency, just as proposed in [9]. In this work, the vital capacity (VC) assessment was conducted with the TPs-based sensor. As the water vapor in breath would have an effect on the response as will be discussed later, the breath air went through a conical flask containing water before it reached the sensor to avoid the influence on the VC test due to the humidity differences for different subjects' breaths. Figure 4a shows the test scheme. The setup was kept unchanged except for the mouthpieces for different subjects. Figure 4b–d shows the variations of TP frequency with four times of consecutive VC test for three subjects. The area enclosed by the curve and the base characterizes the VC. The mean area for each subject was calculated and approximately equaled to 1.09e3, 8.95e2, and 1.36e3 for the subjects No. 1, No. 2, and No. 3, respectively.

### 3.3. Effects of Breath Airflow and Water Vapor

**Figure 5.**
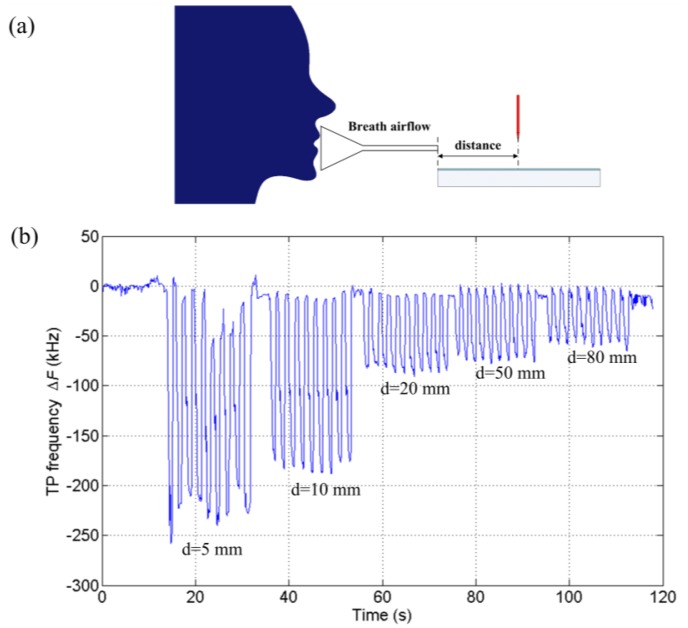
(**a**) Experiment scheme for the effect of airflow; (**b**) Variations of TP frequency with different distances from the needle tip.

The effectiveness of TPs for respiratory monitoring has been demonstrated as shown above and the effects of breath airflow and water vapor in breath are investigated in this subsection. Airflow would have an impact on the TP frequency and make it tend to decrease [31,33]. Generally, the larger the flow velocity is, the more the TP frequency reduces. To validate this, experiments were performed. The breath air velocity was adjusted by changing the distance between the gas tube and the needle as is shown in Figure 5a. The subjects took steady breaths continually with changing distances. Figure 5b illustrates the variations of TP frequency with different distances. Apparently, the magnitude of change decreases with increasing distance. It was also found that only the airflow in the limited discharge area close to the needle tip would effectively impact the TP frequency. If the direction of the small gas tube shifted from the symmetry axis between the needle and the plate, the effect of airflow would be weak. That is to say, the turbulence in ambient air or airflows outside the discharge gap will not influence the response of the TPs-based sensor. It only requires the breath airflow act on the small discharge area.

**Figure 6.**
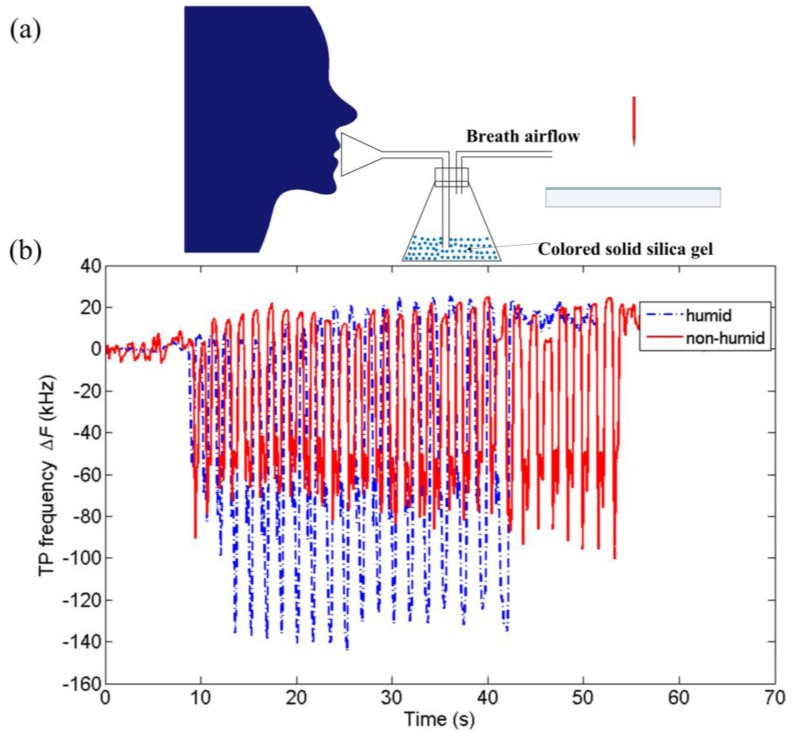
(**a**) Experiment scheme for the effect of water vapor; (**b**) Variations of TP frequency with humid and non-humid breath air.

It was found that the breath airflow did not fully account for the high sensitivity of the TPs-based sensor. The TP frequency would not keep decreasing with increasing flow velocity but rather tend to be unchanged as shown in [31]. The chemical composition, e.g., the water vapor, in breath may also have an effect on the change of TP frequency. In this work, the effect of water vapor was studied by changing the humidity of breath air. The breath air went through a conical flask containing colored solid silica gel before it reached the sensor as shown in Figure 6a. To compare the difference between humid and non-humid breath air and avoid the influence of other factors, e.g., the flow velocity, the experiment for dry breath air was taken with unused colored solid silica gel absorbing the water vapor while the experiment for humid breath air was taken with the same amount of saturated solid silica gel. The same subject was required to participate in the both experiments with normal steady respiration. The test results are shown in Figure 6b. Obviously, the water vapor in breath air reduces the TP frequency significantly. The water containing in the exhaled flow was in atomized state [34]. This is different from the water in ambient air and of great importance for the response of the TPs-based sensor. It will be shown later that the humidity of ambient air has a totally reversed impact on the TP frequency. Moreover, since many volatile compounds and even charged particles are also included in the exhaled flow [34], they may also have some effects. This may lead to electrical respiration sensing of both dynamic and chemical characteristics in a single sensor, which is our future interest and needs more work.

### 3.4. Effects of Applied Voltage and Ambient Parameters

As mentioned above, the application of TPs for respiratory monitoring depends on the change in TP frequency and the baseline frequency does not matter very much. However, the effects of other parameters on the baseline are of importance for the configuration of practical application as well as the understanding of the phenomenon.

#### 3.4.1. Effect of Applied Voltage on TPs

**Figure 7.**
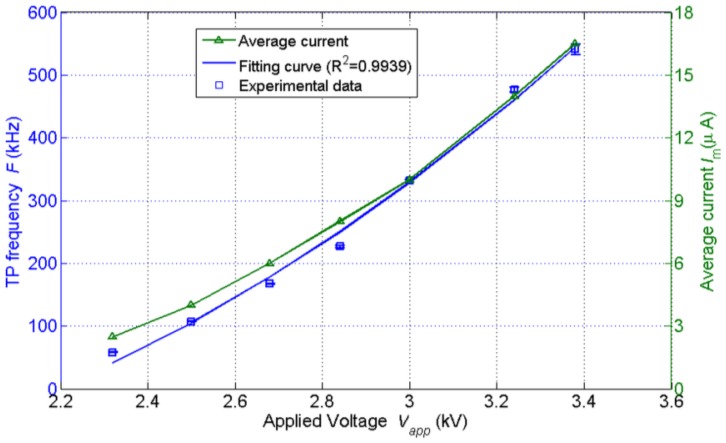
Variations of TP frequency and average current with applied voltage.

Systematic experimental studies have been done previously [35]. In Reference [35], the effects of curvature of needle, gap spacing, and applied voltage on the TPs were investigated. The TP frequency was a function of these parameters and the relationship could be analytically expressed as follows [35]:

(1) $$F=kV_{\text{app}}(V_{\text{app}}-V_{0})/rs^{2}$$

where the *F* is the TP frequency (kHz), *k* a constant coefficient, *V*_app_ voltage applied to the needle electrode (kV), *V*_0_ the threshold voltage (kV), *r* the needle tip radius (mm) and *s* the gap spacing (mm). In our experiments, the impact of applied voltage was investigated by changing the applied voltage whereas other parameters were kept unchanged. The variation of TP frequency with applied voltage is shown in Figure 7. A least-square-fit was performed on the experimental data and the expression is:

(2) $$F=135V_{\text{app}}(V_{\text{app}}-2.2)$$

As can be seen, when other parameters, such as gap spacing and needle radius, are constant, the TP frequency increases with applied voltage in quadratic form. The average current increases with applied voltage roughly in the same way and it falls in the range of from a few to dozens of μA as is shown in Figure 7. The effect of applied voltage is easy to understand. The higher the applied voltage is, the stronger the electric field is. As a result, the ionization is more intense and the drift velocities of negative ions are bigger. Thus, the TP frequency increases with applied voltage as well as the average current.

Generally, when the applied voltage is close to, as well as far beyond, the threshold, the stability of TPs deteriorates. As a matter of fact, when the applied voltage is not high enough, the regular TPs will not build up. On the other hand, when the applied voltage is excessively high and reaches another threshold for discharge transition, the TPs will disappear and the current becomes steady instead of a pulsating one. Keeping this in mind is meaningful for the configuration of a TPs-based sensor. Moreover, it seems that the requirement of high voltage for the sensor is an obstacle for the application. This problem is not difficult to resolve. Some methods can be used to reduce the threshold for TPs, such as smaller gap spacing, smaller tip radius, and even super tip manufactured by micro-nano technology. It can be seen from Equation (1), the radius of the needle tip is of great significance for the TPs. The smaller the radius was, the higher the TP frequency was, as demonstrated in [35]. Apparently, a smaller tip leads to a more intense electric field near the tip with the same applied voltage. In other words, the threshold for ionization as well as the TPs could be reduced by reducing the needle tip diameter. For instance, CNTs and metallic nanowires have been used to reduce the breakdown voltage down to dozens of volts [20–22]. Thus, the structure is very simple and flexible and there is no rigorous demand for the sensor. The construction of low-voltage and miniaturized TPs-based sensor is promising, which is our planning work.

#### 3.4.2. Effects of Temperature and Relative Humidity

**Figure 8.**
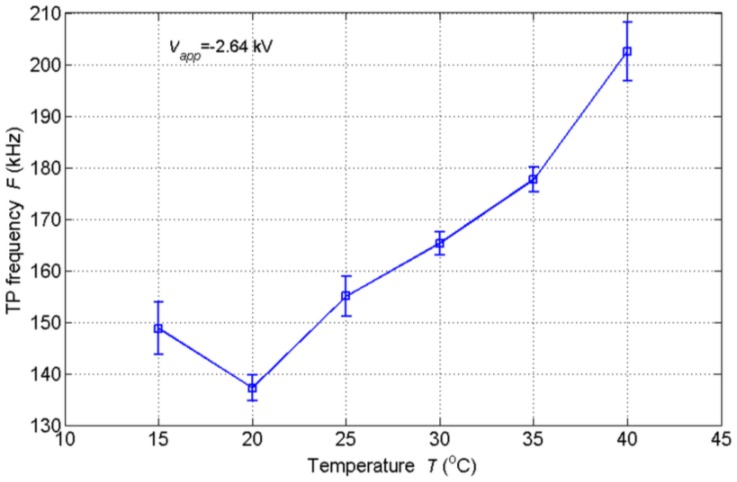
Variation of TP frequency with ambient temperature.

Since the sensor worked in open air in the above experiments, the changes in ambient temperature and relative humidity may have an influence on the TP frequency. Experiments were also conducted to investigate the influences. The NTP was placed in a temperature/humidity chamber (SH-261, ESPEC, Osaka, Japan) which could provide specific temperature and relative humidity environment. In the temperature experiments, the temperature varied from 15 °C to 40 °C with the relative humidity being uncontrolled. The applied voltage was −2.64 kV and the other parameters were the same as above. Figure 8 shows the variation of TP frequency with temperature. In general, the TP frequency increases with temperature. When the temperature is relatively low, the TP frequency shows small changes. However, when the temperature is relatively high (over 30 °C), the changes become bigger. The diffusions of molecules as well as the ions speed up with increasing temperature. The movement of negative ions is faster and leads a higher TP frequency at a higher temperature. However, the movements of negative ions consist of diffusion and drift components. The former is related to the thermal motion and concentration gradient while the latter is driven by the electric field which is much faster. Thus, the influence of temperature is not great.

**Figure 9.**
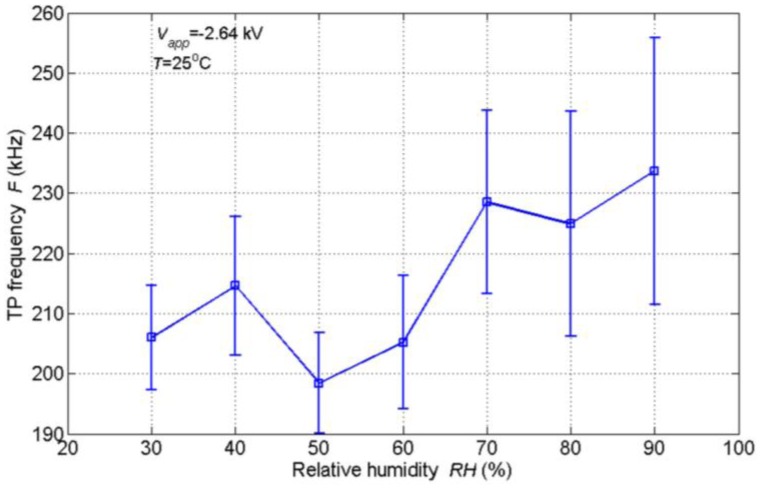
Variation of TP frequency with ambient relative humidity.

In the relative humidity experiments, the temperature was set to 25 °C and the relative humidity changed from 30% to 90%. The other parameters were kept unchanged. The variation of TP frequency with relative humidity is demonstrated in Figure 9. It can be noted that the TP frequency doesn't change a lot with increasing humidity. This is totally different from the above respiration experiments. It is supposed that the state of the water molecules accounts for the discrepancy. As previously mentioned, the water in the exhaled flow is in an atomized state, while the water in ambient air is mainly in a molecular state. The increase of water molecules in air doesn't have a great influence on the formations and movements of negative ions. Moreover, the stability of TP frequency is not good as can be seen in Figure 9. It is found that the interferences introduced by the chamber, e.g., the mechanical vibration and the turbulence due to the air fan, play a part in the deterioration of stability. However, the comprehensive effect of humidity on the discharge is complicated and remains unclear [36].

On the one hand, the ambient temperature and relative humidity are relatively stable or at least slowly changing variables in the home. On the other hand, their influences are not very strong and opposite in the trend compared with those of respiration. Furthermore, as mentioned earlier, the TPs-based sensor works on the change of TP frequency. In other words, the respiration could be monitored by detecting the signal of the first derivative for the TP frequency, just as proposed in [9]. Therefore, the baseline is not very important. The ambient parameters would not have great influence on the application of the sensor.

## 4. Conclusions

In this paper, we proposed a novel sensor based on TPs for respiratory monitoring. The construction of the sensor was very simple. Only a common sharp electrode and a plate electrode with a constant voltage applied to them were required. Assessments of vital sign parameters, respiratory rates of different patterns and vital capacity, were achieved with a simple experimental system. The respiration information could be conveniently extracted by detecting the changes in TP frequency. The shift frequency was from dozens to hundreds of kHz and the ratio of the shift frequency to the baseline could be from 20% to over 90%. It was found that the breath airflow and the atomized water in exhaled air accounted for the high sensitivity of the sensor. The effects of applied voltage and ambient temperature and humidity were investigated. These parameters could change the baseline TP frequency but not influence the application of sensor very much.

The proposed sensor works on the field ionization and the dynamics of charged particles in a short gap. The response is very fast since the ionization process and the transportation for charged particles develop rapidly. Although the sensor has advantages of high sensitivity, simplicity and fast response, its disadvantage may be its requirement for high voltage. However, the sensor performs near the onset of discharge which is weak. The threshold could be lowered by some techniques such as smaller gap spacing, smaller radius of tip and super tip manufactured by micro-nano technology. The miniaturization of the sensor is quite possible and is the goal of our future work. Thus, the respiration sensor based on the proposed method is very promising to be widely applied in home healthcare.
